# Device's design and clinical perspectives for resistant hypertension therapy

**DOI:** 10.1016/j.ijcrp.2024.200240

**Published:** 2024-01-23

**Authors:** Oussama Jami, El Allam Oussama, Zaki Mohammed, Imai Soulaymane, Ben Sahi Ilhaam, Youssef Tijani, Ettahir Aziz

**Affiliations:** a Mohammed V University in Rabat, High School of Technology in Salé; Materials, Energy and Acoustics Team, Rabat, Morocco; b Mohammed VI University of Health Sciences, Biomedical Engineering Department, Casablanca, Morocco; c National High School of Arts and Crafts of Casablanca, Hassan II University of Casablanca, Morocco; d Mohammed VI University of Health Sciences, Faculty of Medicine, Casablanca, Morocco

**Keywords:** Device-based therapy, Secondary hypertension, Sympathetic nerve activity, Device's design, Clinical trials

## Abstract

**Introduction:**

Hypertension is the leading cause of death in the cardiovascular system. Indeed, untreated hypertension can affect one's general health, but medicine can help hypertensive people reduce their chance of developing high blood pressure. However, secondary hypertension remains an unresolved illness.

**Areas covered:**

This review will go through the typical and unusual device-based therapies for resistant hypertension that have arisen in recent years. Further to that, the innovations developed in device-based RH treatment will be covered, as well as the research and studies assessing these novel technologies.

**Expert opinion:**

The innovative device-based techniques that target resistant hypertension provide a potential therapy that has been backed by a number of studies and clinical trials, whereas pharmacological non-adherence and increased sympathetic activity are recognized to be the primary causes of resistant hypertension. Nevertheless, some limitations will be critical for the future of these RH systems, with the device's design and larger RCTs playing a significant role in determining whether a position in routine treatment could be warranted.

## Abbreviations list:

ANS: Autonomic nervous system
BAT: Baroreflex activation therapy
BP: Blood Pressure
CB: Carotid Body
CNS: Central Nervous System
CNT: Cardiac Neuromodulation Therapy
CPAP: Continuous Positive Airway Pressure
DBP: Diastolic Blood Pressure
ESH: European Society of Hypertension
HIRREM: High-resolution, relational, resonance-based, electro-encephalic mirroring
IDE: Investigational device exemption
MAP: Mean Arterial Pressure
MSCS: Magnetic Stimulation of Carotid Sinus
OSP: Obstructive Sleep Apnea
SBP: Systolic Blood Pressure
RH: Resistant Hypertension
RHT: Resistant Hypertension therapy
SNS: Sympathetic Nervous System
TENS: Transcutaneous Electrical Nerve Stimulation

## 1 Introduction

The American College of Cardiology and the American Heart Association (ACC/AHA) define RH as uncontrolled BP level while using 3 different classes of medication and one diuretic [1,2]. Moreover, the two associations recently issued revised recommendations that make the control target of BP < 130/80 mmHg in systole and diastole, respectively, which increase more the prevalence of RH [3].

On the other hand, the European Society of Hypertension (ESH) has also provided their perspective on the appropriate characterization of resistant hypertension (RH) through their recently published guidelines. Specifically, the ESH delineates RH as the failure to lower office BP to <140/90 mmHg despite receiving at least three concurrent antihypertensive drug classes, ideally including a diuretic. Furthermore, the ESH position specifies that uncontrolled 24-h BP levels (130 mmHg SBP or 80 mmHg DBP) should be confirmed by out-of-office BP measurements [4,5].

After eliminating pseudo-hypertension and white-coat hypertension, as well as non-adherence, the RH is classified as true. As recommended [6], optimizing the drug regimen, making lifestyle changes, and adding second-line antihypertensive medication could lead to a significant improvement, while a secondary hypertension evaluation can be conducted when RH is still unable to meet the recommended blood pressure threshold; device-based therapy will then be considered.

Nowadays, fourteen device-based treatments are well developed, either technically or in clinical studies, especially BAT and RDN, which are on their way to being utilized in routine treatment. Indeed, the new ESH guidelines also highlighted the importance of RDN as novel treatment approaches and ESH focuses on device-based therapy in HTN from a variety of angles, with the objective of lowering HTN morbidity through ESH Excellent Centres, notably the ESH Interventional Treatment of Hypertension Working Group.

This article will scrutinize RH devices and their specifications, present an overview of the clinical trials and studies available for each therapy, and compare RH devices in a single table that contains the most mature devices and summarizes special characteristics and current status.

## 2 Renal denervation

In this section, we will not go into detail about RDN, but we will examine the key stages that determined the evolution of this treatment for the following reasons: RDN is currently the most developed RH treatment, having previously demonstrated its viability in several studies, including large RCTs. Furthermore, considerable research has been done in the literature, which has investigated this therapy significantly and more thoroughly than the other therapies. [[7], [8], [9], [10], [11], [12], [13]], showing an overall coverage of more than the half of RH therapy. In addition, more than 10 RDN systems have been given (Seen Table 1) have received clinical approval and the CE mark.

**Table 1:**
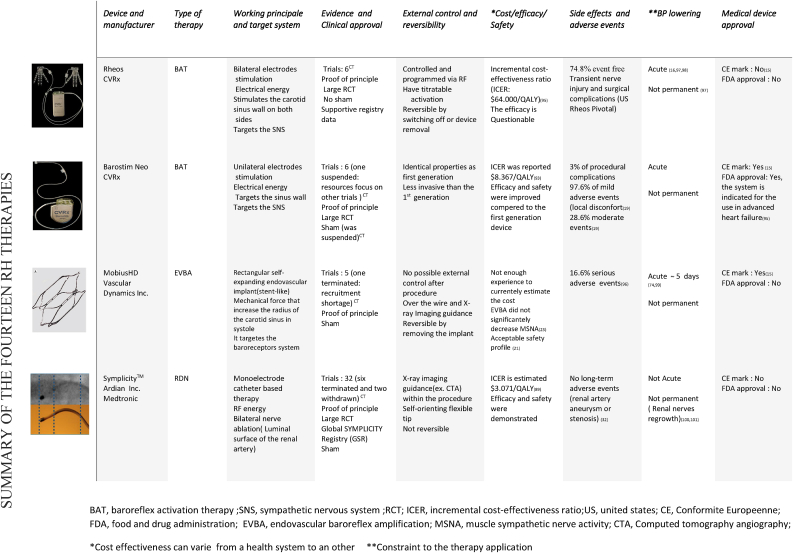

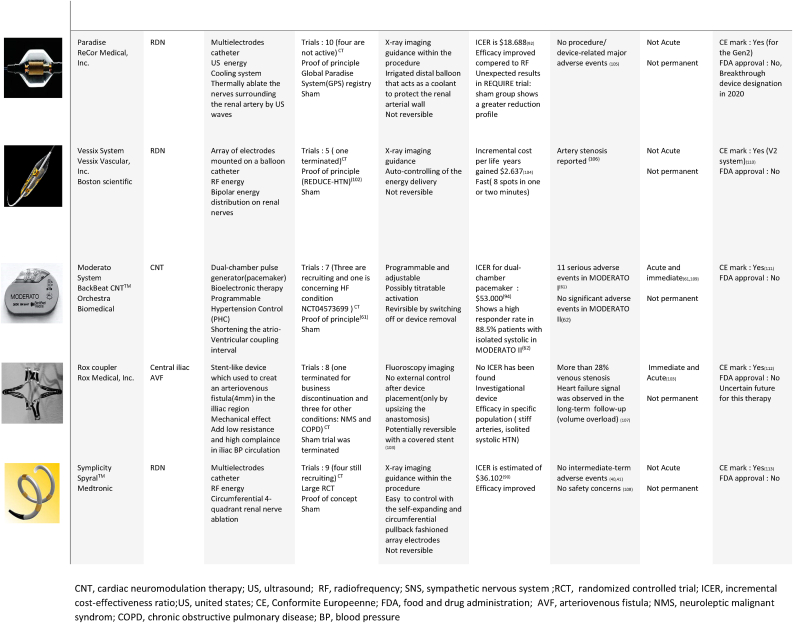

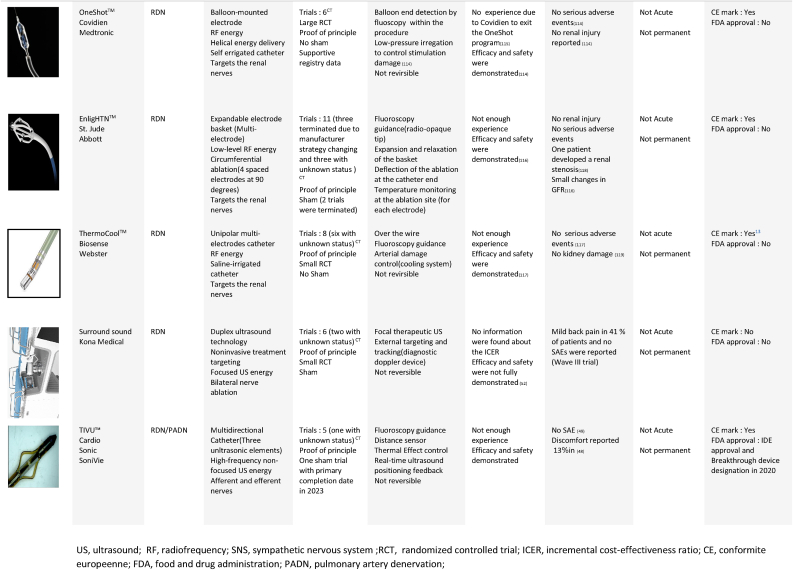
RHT related devices. [[14], [15], [16], [17], [18], [19], [20], [21], [22], [23], [24], [25], [26], [27], [28], [29], [30], [31], [32], [33], [34], [35], [36], [37]]

BAT, baroreflex activation therapy; SNS, sympathetic nervous system; RCT; ICER, incremental cost-effectiveness ratio; US, united states; CE, Conformite Europeenne; FDA, food and drug administration; EVBA, endovascular baroreflex amplification; MSNA, muscle sympathetic nerve activity; CTA, Computed tomography angiography.

*Cost effectiveness can varie from a health system to an other **Constraint to the therapy application.

CNT, cardiac neuromodulation therapy; US, ultrasound; RF, radiofrequency; SNS, sympathetic nervous system; RCT, randomized controlled trial; ICER, incremental cost-effectiveness ratio; US, united states; CE, Conformite Europeenne; FDA, food and drug administration; AVF, arteriovenous fistula; NMS, neuroleptic malignant syndrom; COPD, chronic obstructive pulmonary disease; BP, blood pressure.

US, ultrasound; RF, radiofrequency; SNS, sympathetic nervous system; RCT, randomized controlled trial; ICER, incremental cost-effectiveness ratio; CE, conformite europeenne; FDA, food and drug administration; PADN, pulmonary artery denervation.

Renal nerves are an important component of kidney functioning and blood pressure management [38]. As a result, they are intensively investigated, particularly the efferent nerves, that thought to play an important role in blood pressure regulation in general including vasoconstriction [39]. RDN has been shown to reduce blood pressure in animals [40,41] and Human subjects [[42], [43], [44]]. Furthermore, numerous approaches have been proposed to obtain effective RDN treatment for greater nerve activity suppression [45,46].

It is well known that SYMPLICITY HTN-1 and SYMPLICITY HTN-2 provide positive findings, which have motivated researchers to more study this innovative approach [45,47], however it fails in the third trial, SYMPLICITY HTN-3, which produced less promising results [48], This finding caught the attention of certain experts, who questioned various trial characteristics [49,50], which ended with DENERHTN trial [51] emphasizing the relevance of trial design while employing the identical mono-electrode radiofrequency electrode [49] SYMPLICITY catheter (see Table 1).

A new generation of catheter-based RDN electrodes, including multi-electrode RF denervation and US denervation, has been added to the RDN device arsenal. Both have been studied in various RCTs, most notably SPYRAL HTN [52,53] and RADIANCE-HTN [[54], [55], [56]]. All trials demonstrated that the RDN group outperformed the sham group. Furthermore, alcohol-mediated denervation and cryo-RDN are being studied to demonstrate their short-term efficacy and safety, and initial data demonstrate that the two systems have a high potential [46,[57], [58], [59]], whilst high-frequency non-focused US was studied in RDN and PADN with the TIVUS system, which got FDA approval, demonstrating good safety and efficacy results [60,61] (see Table 1).

To conclude, RDN is the most established device-based therapy, with healthcare practitioners, the biomedical sector, and the research community all taking note of its relevant outcomes and bright future. Given these benefits, it is nevertheless recommended that this treatment should not be used outside of clinical trials until sufficient evidence is gathered [1]. Furthermore, as of late 2022, RDN has not received US approval.

## 3 Baroreflex amplification

The Baroreflex Activation Therapy (BAT) is a cutting-edge and potential device-based treatment for secondary hypertension that uses the baroreceptor reflex system to adjust ANS activity and regulate blood pressure [62]. In early tests, this procedure yielded promising results, but it was discontinued due to technological and safety risks [63]. After encouraging results demonstrating the influence of electrical impulses of the carotid body on blood pressure in animals [64,65] and humans [66], a device was developed. With the development era of modern device-based treatments starting from the beginning of the 21st century, and with the technical breakthroughs possessed by CVRx [67], this device was revived; additional information about the two generations of CVRx is illustrated in Table 1.

Clinical data were provided in clinical trials' publications underpinning the efficacy and safety of the two CVRx generations: The Rheos Pivotal trial [68], which was the first trial for the first-generation but was halted, and the Barostim Neo Trial [69], which was for the second-generation, which recently have got the FDA approval for heart failure. To this day, 12 studies for RH condition were registered at the “clinical trials” database (see Table 1), along with ten 10 clinical trials for heart failure, importantly was the relevant effectiveness of baroreflex activation, despite the fact that some endpoints was not met in some study results [68], baroreflex activation therapy has several advantages above other devices of which the quick conversion of this therapy helps discriminate between real responders and patients with less favorable treatment results.

To confront some of the limitations of this device, development of a new design has been made [7], a baroreflex activation that will decrease the invasiveness, resolve the battery management concern, and because baroreflex continuously disrupted in hypertension, where it decreases heart rate instantly after rapid blood pressure increase. Aligned with that proposal, we wanted to integrate a system that may overcome these weaknesses and prevent some adverse effects, as well as reduce stimulation-related side effects, which can occur even with the latest generation [70,71]. The goal of this novel technique is to design an external prototype that uses a wireless energy transmission module to precisely stimulate the baroreflex area. This new BAT design will be extremely beneficial since it will keep just the internal system will be attached to the carotid artery. A proof-of-concept study is carried out establishing the feasibility of this novel BAT technique, and the details of the results will be published soon.

## 4 Endovascular baroreflex amplification

Endovascular baroreflex amplification (EVBA) is a minimally invasive device that regulates the baroreceptors by mechanically expanding/contracting the carotid sinus wall, resulting in increased sympathetic activity and, ultimately, decreased blood pressure. Vascular Dynamics introduced two devices that may be implanted intravascularly and extravascularly in the carotid sinus semi-invasively.

Clinical data revealed the efficacy of EVBA, as compared to a self-expanding stent, the MobiusHD device demonstrated an immediate and sustained BP reduction as well as a higher safety profile [72]. The open-label, multicenter, first-in-man clinical trial CALM-FIM (NCT01911897)^CT^ was completed in May 2013, with data showing long-term events included two strokes and one ischemic attack at two years [70]. Moreover, a substantial reduction in Office BP was recorded after 6 months, with drops of 24 in systolic BP and 12 mm Hg in diastolic BP [72]. After three years of follow-up, the Systolic BP had dropped by 30 mm Hg, proving that EVBA is effective at lowering blood pressure and has a tolerable risk characteristic [73].

In parallel, two clinical trials (NCT03179800 and NCT02827032, respectively)^CT^ are evaluating the function of MobiusHD in decreasing BP levels while maintaining an acceptable safety profile. More randomized sham-controlled trials are required to demonstrate the effectiveness and safety of this approach, in which all medical teams collaborate to ensure the success of the MobiusHD device [74].

## 5 Arteriovenous anastomosis

Arteriovenous Anastomosis Fistula (AVF) is the single device that treats the hemodynamic components of the blood pressure with no need for an intermediary and has a directly and immediately influence on the RH. The AVF is a stent-like device used to create a 4 mm arteriovenous fistula in the illiac area which applies a mechanical force to add low resistance and high compliance in the iliac BP circulation. The most well-known AVF device is the ROX Coupler, which was approved to reduce blood pressure immediately after an AVF surgery [75]. Inspite of its effectiveness in decreasing BP and the controllable venous stenosis events that represent more than 25 % of patients, the CONTROL HTN-2 trail was halted early due to Heart Failure concerns [76], thereby ending this innovative therapy.

Since the issues include venous stenosis and heart failure, the first of which is treatable, this device may be revived if we tried to reduce the adverse events by designing a new safe system and an appropriate therapeutic surgery based on the same blood pressure reduction concept. This technique can be kept to a minimum risk by employing a device that controls systemic circulation while leaving cardiac variabilities intact.

## 6 Pacemaker‐Based cardiac neuromodulation

More than 70 % of pacemaker patients have hypertension [77], which is one of the main reasons that cardiac neuromodulation therapy (CNT) was proposed for blood pressure regulation. In this regard, the BackBeat Moderato system, a dual-chamber pulse generator was introduced which can modify cardiac output by modulating the atrioventricular intervals, reducing LV loading and thus lowering blood pressure [78].

BackBeat Moderato was assessed in two randomized clinical trials, MODERATO-I and MODERATO-II (NCT02282033 and NCT02837445, respectively)^CT^ [78,79]. The first study involved 35 participants who had their SBP less than 20 mmHg after 3 months, whereas the second sham study enrolled 68 participants (47 met the trial criteria) who had their SBP less than 11 mmHg after 6 months. MODERATO-I had a few adverse events, however MODERATO-II had no notable adverse events.

This therapy may be safe and effective for persons with resistant hypertension, according to the evidence. Furthermore, doctors anticipate a bright future for this treatment, with four studies planned to be finished by 2023 to further assess the long-term efficay and safety. Yet, there are also limits in terms of long-term safety concerns and whether individuals without pacemaker indications might use this device due to its high cost [80].

## 7 Other device-based therapies used for resistant hypertension

Additionally, to the above-mentioned device-based therapies for RH, several BP-lowering device approaches are aiming to take their place in the armamentarium of RH-approved devices. Some of these are in advanced clinical trials, while others are being discontinued or withdrawn for reasons that are not necessarily related to efficacy or safety concerns.

Starting with the devices that've either been through clinical trials or been registered in a database of clinical trials. Transcutaneous Electrical Nerve Stimulation (TENS) is a neuro-modulatory device widely used in neuro-modulation therapy, especially in pain relief, and evidence shows that the application of TENS may have positive results in treating resistant hypertension. Effectiveness will be evaluated with a randomized sham-controlled trial expected to be completed in June 2022 (NCT02365974) ^CT^, however, no results have been published yet, same clinical trial design was for Transcutaneous Vagal Sensory Stimulation trial, where the data collection will be completed in April 2023 (NCT05179343)^CT^. Although the non-significant BP lowering effect in some studies regardless of the intensity of stimulation [81], recent studies demonstrate that TENS significantly improves BP [[81], [82], [83]] and conventional stimulation in transcutaneous vagus nerve stimulation has a safe profile, as evidenced by a review of 51 studies involving 1322 subjects who received this therapy [84]. Similarly, extracorporeal shock wave therapy is a noninvasive approach that exposes tissues to US to increase their perfusion and promote angiogenesis; an unpublished study attempted to demonstrate its safety and efficacy in the treatment of resistant hypertension using a shock wave of 0.09–0.1 mJ/mm2 (NCT02042066) ^CT^.

Another interesting approach for carotid sinus modulation is the Magnetic Stimulation of Carotid Sinus (MSCS), evaluation in rabbit models has demonstrated a 10.4 ± 2.3 mmHg decrease in MAP [85], but the concept of a wearable device is not yet a practical idea for the MSCS technology.

A small randomized controlled trial of a High-resolution, relational, resonance-based, electro-encephalic mirroring (HIRREM) system for resistant hypertension is underway, with results still pending (NCT03479697).

According to several studies, the efficacy of Continuous Positive Airway Pressure (CPAP), a widely used device in Obstructive Sleep Apnea (OSA), was demonstrated in both RH and OSA [[85], [86], [87]]. In a randomized clinical trial (NCT00616265) ^CT^ the decrease was 3.1 mm Hg in mean BP and a significant improvement in nocturnal BP was reported [[86], [87]], a reduction in aldosterone excretion was also reported in optimal CPAP treatment [88]. However, later studies reported no significant effect on daytime BP in resistant hypertension compared with the important reduction in nocturnal BP which the measured fall was of 2.2 % [89], another clinical trial enrolling 1371 participants showed similar BP response (NCT03002558) ^CT^ and an average nighttime BP reported to be 5.72 mm Hg [90], but more results are expected from this trial, with the estimated study completion is June 2023.

It is worth recalling that RDN can be performed with other different approaches, for example, alcohol injection, cryoenergy ablation, and external irradiation with ultrasound low-frequency waves, or small amounts of radiation delivered to the treatment zone with the Beta-Cath 3.5 F system. The latter showed a significant decrease in SBP of more than 10 mmHg at 6 months following the procedure (NCT01968785) ^CT^, but was terminated due to safety concerns.

Other devices were tested in trials, but they didn't achieve the efficacy outcome or received little attention from researchers. For instance, Enhanced External Counter Pulsation (EECP), Cryoenergy renal ablation, etc.

## 8 Conclusion and opinion

It is crystal clear that the development of device-based therapy for RH has progressed exponentially in recent years using several approaches that target different structures and organs; each differs in efficacy and safety profile. As a result, the future of each device will be settled by the long-term results of RCTs (10-plus years). Nevertheless, efficacy and safety should be correlated with the blood pressure control recommendations. In this regard, we want to emphasize the importance of the noninvasive approaches in RH device-based therapy; in particular, we have proposed a new approach for BAT with a less invasive procedure and a more patient-oriented design for specific BP control; the proposal is now in an advanced stage of prototyping, with results expected in the next weeks.

We covered the most recent technologies and clinical studies for device-based therapy in the RH condition, as well as a summary of fourteen device-based therapies covering both technological and clinical characteristics (Table 1), to demonstrate the potential of this novel therapy. Furthermore, we highlighted some of the limits of existing therapies, as well as brief proposals for overcoming some of these hurdles. In addition, if these therapies could make it in larger RCTs by producing sufficient results and perfect precision in subject stratification that specifies eligibility profiles to optimize the advantages, in a few years, device-based therapy in RH may evolve into a regular therapeutic treatment.

## Declarations

No conflicts of interest to disclose.

No experiments have been carried out involving animal or human subjects.

## Limitation

The main constraints of this review are COVID-19 pandemic as well as certain restrictions on the availability of data, notably for clinical studies, and the short time span in which thorough information was gathered.

## CRediT authorship contribution statement

**Oussama Jami:** Conceptualization, Writing – original draft, Writing – review & editing, Data curation, Formal analysis, Investigation, Methodology, Project administration, Resources, Validation. **El Allam Oussama:** Investigation. **Zaki Mohammed:** Formal analysis, Investigation. **Imai Soulaymane:** Writing – original draft, Writing – review & editing. **Ben Sahi Ilhaam:** Investigation. **Youssef Tijani:** Investigation, Validation. **Ettahir Aziz:** Methodology, Supervision.
